# Bridging knowledge gaps: a bibliometric analysis of non-invasive ventilation in palliative care studies

**DOI:** 10.1186/s44158-024-00140-y

**Published:** 2024-01-24

**Authors:** Marco Cascella, Federica Monaco, Alessandro Vittori, Mostafa Elshazly, Annalisa Carlucci, Ornella Piazza

**Affiliations:** 1 Department of Medicine, Surgery and Dentistry, University of Salerno, 84081 Baronissi, Italy; 2 Unit of Anesthesia, ASL NA/1, Naples, Italy; 3 Department of Anesthesia and Critical Care, ARCO ROMA, Ospedale Pediatrico Bambino Gesù IRCCS, 00165 Rome, Italy; 4 Respiratory Critical Care, Cairo University, Cairo, Egypt; 5 Pulmonary Rehabilitation Unit, Istituti Clinici Scientifici Maugeri, Pavia, Italy

**Keywords:** Non-invasive ventilation, Palliative care, Bibliometrics, VOSviewer, CiteSpace, Cancer, Amyotrophic lateral sclerosis, Specialized palliative care

## Abstract

**Background:**

Despite being a useful strategy for providing respiratory support to patients with advanced or terminal illnesses, non-invasive ventilation (NIV) requires in-depth investigation in several key aspects.

**Objectives:**

This bibliometric analysis seeks to comprehensively examine the existing research on the subject. Its goal is to uncover valuable insights that can inform the prediction trajectory of studies, guide the implementation of corrective measures, and contribute to the improvement of research networks.

**Methods:**

A comprehensive review of literature on NIV in the context of palliative care was conducted using the Web of Science core collection online database. The search utilized the key terms “non-invasive ventilation” and “palliative care” to identify the most relevant articles. All data were gathered on November 7, 2023. Relevant information from documents meeting the specified criteria was extracted, and Journal Citation Reports™ 2022 (Clarivate Analytics) served as the data source. The analysis employed literature analysis and knowledge visualization tools, specifically CiteScope (version 6.2.R4) and VOSviewer (version 1.6.20).

**Results:**

A dataset with bibliometric findings from 192 items was analyzed. We found a consistent upward of the scientific output trend over time. Guidelines on amyotrophic lateral sclerosis management received the highest number of citations. Most documents were published in top-ranked journals. Less than one-third of the documents pertain to clinical studies, especially retrospective analyses (25%). Key topics such as “decision making”, and “communication” were less addressed.

**Conclusions:**

Given the substantial clinical implications, further high-quality studies on this subject are recommended. Encouraging international collaborations is needed. Despite the growing volume of documents in the field, this bibliometric analysis indicates a decline in collaborative networks.

## Introduction

Palliative care is a multidisciplinary approach aimed at improving the quality of life for patients and their families facing serious, progressive, or terminal illnesses. The primary goal of palliative care is to alleviate pain and other physical discomforts, while also providing emotional, social, and spiritual support [1]. According to the recommendations of the European Association for Palliative Care (EAPC), advanced palliative care is provided through specialized services for patients dealing with complex issues that are not adequately addressed by other treatment options. These highly complex palliative care services necessitate a collaborative approach, integrating a multi-professional team with an interdisciplinary method of operation [2].

Patients with advanced or terminal illnesses, such as cancers [3], end-stage chronic obstructive pulmonary disease (COPD) [4], amyotrophic lateral sclerosis (ALS) [5], or other diseases may experience respiratory distress. In this complex clinical scenario, non-invasive ventilation (NIV) can be implemented to provide respiratory support without the need for invasive interventions including intubation and invasive mechanical ventilation [6]. Therefore, NIV can be employed for specialized palliative care for facing respiratory symptoms and optimizing the patient’s quality of life [7, 8]. This therapeutic approach can foster improved sleep, alleviate anxiety, and enhance comfort, empowering patients to engage more fully in meaningful activities and interactions with their loved ones [9].

Nevertheless, for a deeper comprehension and to refine patient care, it is essential to investigate several crucial aspects of NIV applications in palliative care. For example, the unpredictable nature of these illnesses often complicates the application of prognostication-based strategies. Therefore, Therefore, it is essential to define appropriate criteria for selecting patients who would derive the maximum benefit from NIV [10]. Patient characteristics, such as disease stage, comorbidities, and symptom severity should be carefully evaluated for the success of NIV within a multidisciplinary respiratory care program [11]. Other aspects need to be carefully evaluated. Notably, it becomes necessary to deliver palliative care following patients' needs, and concurrently with disease-modifying therapies. It is mandatory to explore patient acceptance and tolerance of NIV in the context of palliative care and investigate factors that may affect their compliance and comfort. Since integration with palliative care modalities such as pain management, psychological support, and end-of-life discussions can be difficult, precise palliative pathways for identifying potential barriers and facilitators to the incorporation of NIV into palliative care practice are needed [12].

Crucial factors that demand attention include healthcare provider perspectives and caregiver support and education [13] as well as application modalities in particular clinical settings such as intensive care units (ICUs) [14] or young patients [15]. Finally, ethical considerations surrounding the use of NIV in palliative care [16], especially in decision-making, withdrawal, or continuation of NIV support during palliative sedation at the end of life, must be carefully examined [17].

Given these premises, a comprehensive investigation of these aspects can contribute to the refinement of guidelines, improved patient outcomes, and the integration of NIV into holistic palliative care approaches. Different research approaches can be adopted for this purpose. Bibliometric analyses are statistical-based methods useful for the quantitative examination of academic literature. These tools can offer a profound understanding of research trends within specific realms of science and technology. The advantages of these analyses include the ability to identify prolific authors, institutions, and key research topics, providing insights into the evolution of scientific knowledge [18].

This bibliometric analysis aims to examine patterns and trends in research on NIV and palliative care, ultimately identifying prevalent themes, methodologies, and focal points within the literature. Additionally, the analysis aims to explore gaps in current research. The output can be useful for informing future research directions and steering initiatives aimed at elevating the overall quality of research in these fields.

## Methods

### Data collection

The methodology aligns with established practices in bibliometric investigations. In these analyses, a single database is implemented to maintain consistency and precision in the results, avoiding data overlap from different sources [19–22]. A comprehensive examination of global literature concerning NIV in palliative care was conducted using the Web of Science Core Collection (WoSCC) online database. The search employed specific terms and strings to pinpoint closely related articles, with the key elements being “non-invasive ventilation” and “palliative care.” The specific search strategy implemented was (non-invasive ventilation (All Fields) AND palliative care (All Fields)). Language restrictions were intentionally omitted to encompass a broad range of publications. All pertinent data were retrieved on November 7, 2023, and subsequently exported in TXT format of “full records and references” and Microsoft Excel (.xlsx) format for further analysis [23]. Bibliometric indicators including quartile (Q) and impact factor (IF) were retrieved from Journal Citation Reports™ (Clarivate, 2022). The search results underwent screening to evaluate the relevance of topics and identify duplicates.

### Data processing

The quantitative analysis was executed using Citation Report from Clarivate Analytics and two Java-based information visualization tools including VOSviewer (version 1.6.20) and CiteSpace (6.2.R4) to perform network analysis (knowledge mapping). While CiteSpace (Chaomei Chen, Philadelphia, PA, USA) primarily focuses on analyzing citation networks to uncover patterns and trends in scholarly literature, VOSviewer (Leiden University, Leiden, the Netherlands) is more versatile and widely used for creating visual representations of collaboration networks, co-authorship networks, and co-occurrence networks based on keywords or terms. Usually, scientometric investigations implement both tools to gain comprehensive insights into the structure and dynamics of scientific knowledge in a particular field. The synergistic use of both tools is a valuable strategy for capturing the breadth of the phenomenon and understanding its temporal trend [18].

For CiteScope we adopted a time slicing of 1 year from 1997 to 2023. For text processing, we selected title, abstract, author keywords, and keywords plus. For node visualization, we selected country, and keyword to create the co-occurrence map. It was considered a g-index in each slice of 8, and no pruning modality. Other parameters use the system default values. In VOSviewer, we customized the minimum number of documents for nodes based on data visualization requirements, leaving other parameters at default values. Therefore, our examination involved the keywords analysis and the maps of co-citations for countries/regions. Finally, a manual screening process was conducted to extract bibliometric data including Q and IF, and to assess the document types.

## Results

### Analysis of annual publications distribution, citations, and geographic origin

**Fig. 1:**
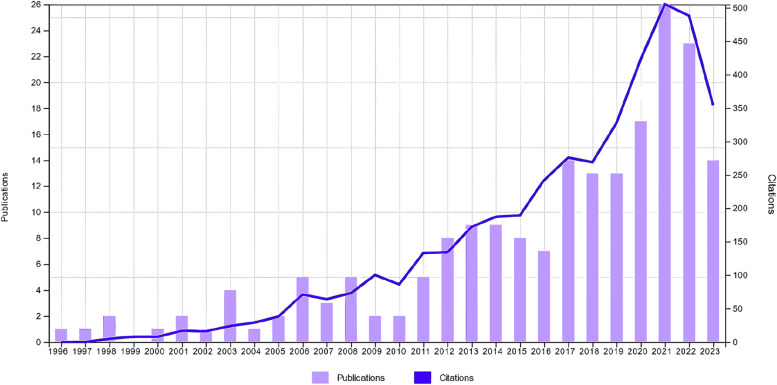
Annual publications and citation distribution (*n* = 192)

By implementing the screening strategy, a total of 198 documents were collected from WoSCC, covering the period from 1996 to 2023. Six items were excluded due to their lack of relevance to the research topics. No duplicates were found. Finally, a set of 192 (174, 87.88% in English) documents was considered for the analyses. According to the Citation Report from WoSCC, the combined total citations for these documents amount to 4240, with an average annual citation frequency of 21.41. There was a consistent upward trend over time (Fig. 1).

The articles garnering the highest number of citations are chiefly centered around the management of ALS. Regarding their typology, they are predominantly guidelines. The 10 most cited documents [24–33] are reported in Table 1.

**Table 1:** Most cited articles

Nr	Title	Journal	Year	Article type	Topic	Cit	Ref
1	EFNS guidelines on the Clinical Management of Amyotrophic Lateral Sclerosis (MALS)-revised report of an EFNS task force	*European Journal of Neurology*	2012	Guideline	Amyotrophic lateral sclerosis	716	[24]
2	EFNS task force on management of amyotrophic lateral sclerosis: guidelines for diagnosing and clinical care of patients and relatives	*European Journal of Neurology*	2005	Guideline	Amyotrophic lateral sclerosis	202	[25]
3	Quality of life and psychosocial issues in ventilated patients with amyotrophic lateral sclerosis and their caregivers	*Journal of Pain and Symptom Management*	2003	Cross-sectional survey	Amyotrophic lateral sclerosis	147	[26]
4	Palliative use of non-invasive ventilation in end-of-life patients with solid tumours: a randomised feasibility trial	*Lancet Oncol*	2013	Randomized trial	Palliative care in oncology	144	[27]
5	EALSC Working Group. Good practice in the management of amyotrophic lateral sclerosis: clinical guidelines. An evidence-based review with good practice points. EALSC Working Group	*Amyotrophic Lateral Sclerosis*	2007	Guideline	Amyotrophic lateral sclerosis	140	[28]
6	Palliative care in the ICU: relief of pain, dyspnea, and thirst–a report from the IPAL-ICU Advisory Board	*Intensive Care Medicine*	2014	Narrative review	Palliative care in ICU	107	[29]
7	Clinical care of patients with amyotrophic lateral sclerosis	*Lancet Neurology*	2007	Narrative review	Amyotrophic lateral sclerosis	93	[30]
8	Examining the evidence about treatment in ALS/MND	*Amyotroph Lateral Scler Other Motor Neuron Disord*	2001	Narrative review	Amyotrophic lateral sclerosis	92	[31]
9	Discontinuation of mechanical ventilation in patients with amyotrophic lateral sclerosis	*Journal of Neurology*	1998	Case reports	Amyotrophic lateral sclerosis	92	[32]
10	Noninvasive Mechanical Ventilation in Acute Respiratory Failure Clinical Practice Guidelines on behalf of the German Society of Pneumology and Ventilatory Medicine	*Pneumologie*	2015	Guideline	Non-invasive ventilation	81	[33]

**Fig. 2:**
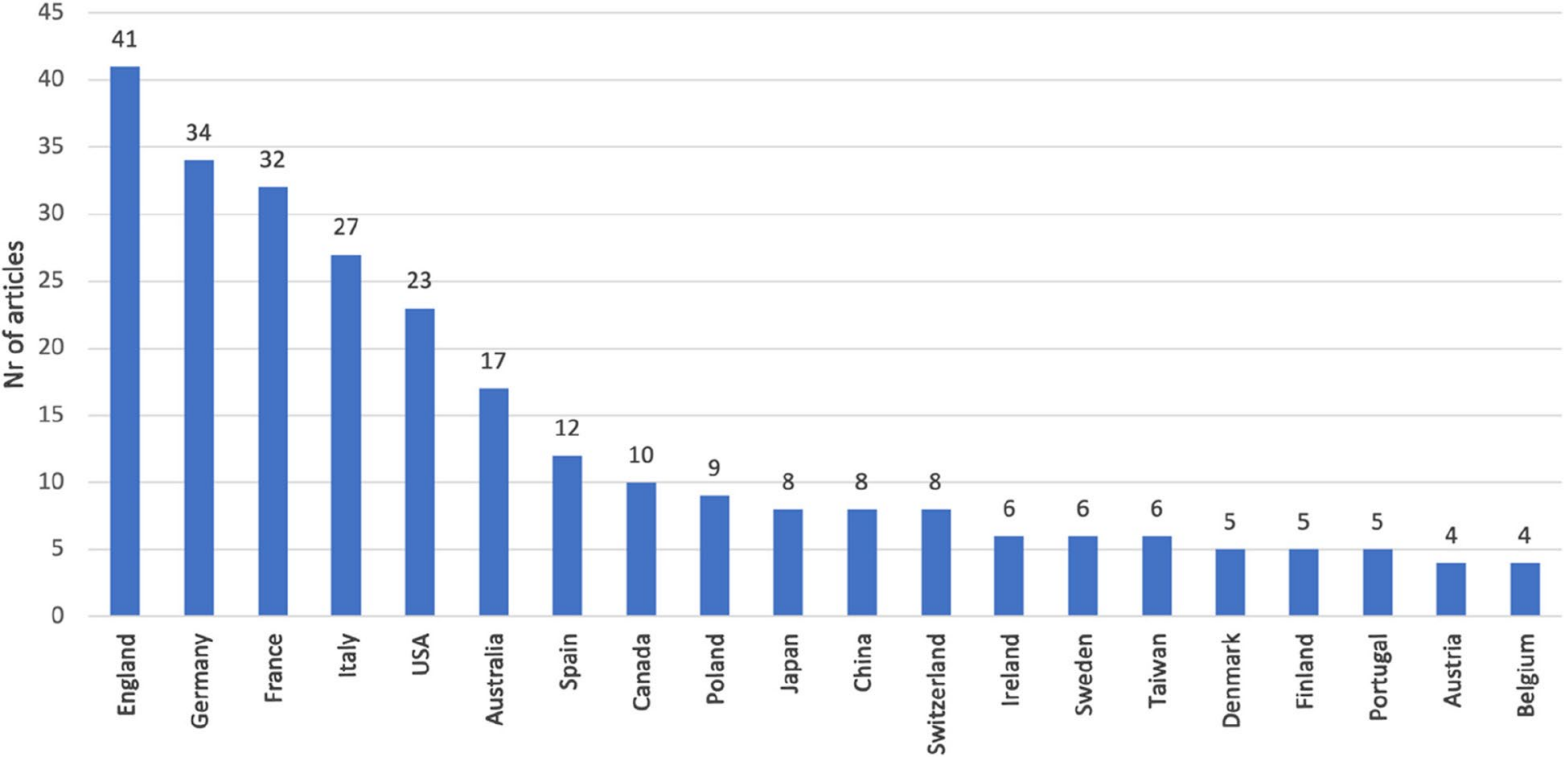
Geographic origin (*n* = 192)

Over half of the documents (107 out of 192) are from Europe, with 41 documents specifically originating from England (Fig. 2).

### Journals

The analysis indicates that these publications are sourced from 113 journals. *Palliative Medicine* tops the list with 12 published documents, *BMJ Supportive & Palliative Care* follows with 9 items, and the *Annals of Palliative Medicine* ranks third with 7 documents. In the whole dataset, 75 documents (39%) were published by journals in Q1; and 56(29%) in Q2. Table 2 enumerates the names of the top 10 journals based on publication frequency; bibliometric indicators are also provided.

**Table 2:** Top 10 journals based on publication frequency

Nr	Journal name	Documents (%)	Impact factor^a^	Category^ab^ quartile
1	*Palliative Medicine*	12(6)	4.4	Q1
2	*BMJ Supportive Palliative Care*	9(4.54)	2.7	Q3
3	*Annals of Palliative Medicine*	7(3.53)	1.9	Q4
4	*BMC Palliative Care*	6(3)	3.1	Q2
5	*Journal of Neurology*	6(3)	6	Q1
6	*Amyotrophic Lateral Sclerosis*	5(2.52)	2.36	Q2
7	*BMJ Open*	5(2.52)	2.9	Q2
8	*Journal of Pain and Symptom Management*	4(2)	4.7	Q1
9	*Amyotrophic Lateral Sclerosis and Frontotemporal Degeneration*	3(1.5)	2.8	Q3
10	*European Respiratory Journal*	3(1.5)	24.9	Q1

*Legend*: ^a^From Journal Citation Reports™ 2022; ^b^For journals included in different categories, the best quartile was used

### Document types

**Fig. 3:**
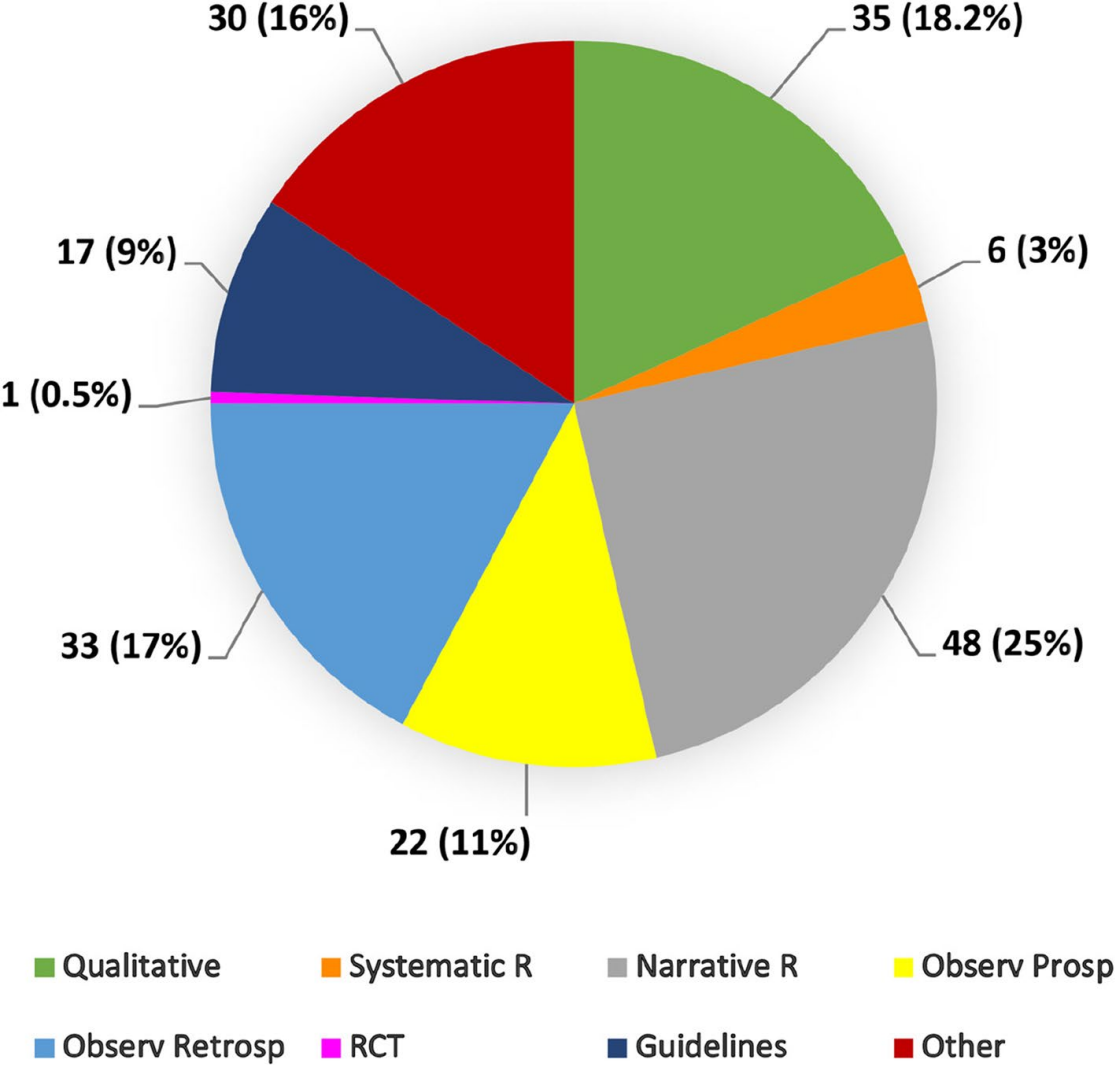
Document types. Qualitative documents include surveys and interviews. The category “Other” includes letters, commentaries, editorials, conference papers, and perspectives

Different types of studies were published. Among the whole set (*n* = 192), we calculated 35 qualitative methodology studies (surveys, interviews); 48 narrative reviews; 22 observational prospective studies; 17 guidelines, and recommendations; and only 1 randomized clinical trial (Fig. 3).

### Bibliometric analysis of the keywords

**Fig. 4:**
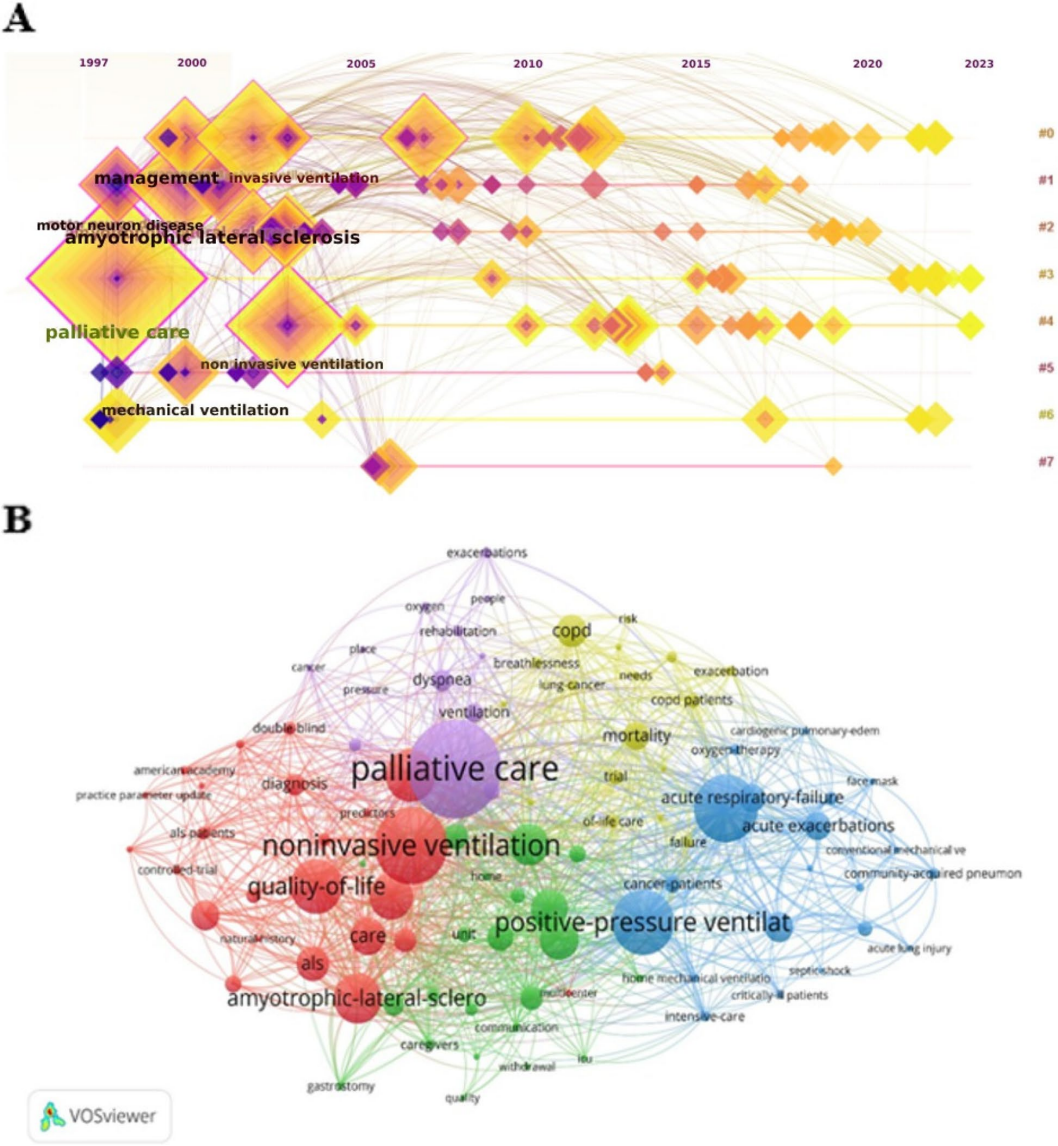
Keywords analysis. The CiteScope analysis showed 8 representative keyword clusters (**A**), structured primarily between 1997 and 2015 and with the keywords “palliative care” and “amyotrophic lateral sclerosis”. For the VOSviewer-based knowledge mapping (**B**) we adopted a minimum number of occurrence keywords of 3; of 462 keywords, 96 reached the threshold; 5 clusters; links 1178; total link strength 1991. The size of nodes reflects the occurrence rate, while the connections (curves) between nodes depict their co-occurrence within a document. A shorter distance between two nodes indicates a higher degree of keyword co-occurrence. Starting from the data entered, the software generates bibliographic maps on its own where in some cases image overlaps are created

The keywords analysis showed 462 items and 5 clusters including nodes of different size (i.e., occurrence rate for keywords). The keyword “palliative care” collected 45 occurrences; the keywords “non-invasive ventilation”, 35; and “decision making”, 11. Key aspects and keywords such as “communication”, “COPD”, “quality of life”, “lung cancer”, “needs”, and methods as well as ventilation strategies encompassing keywords such as “high flow”, and “oxygen therapy” are less addressed. Moreover, connections (i.e., distance between keywords) emphasize the gap in crucial aspects such as links between outcome assessment and ventilation strategies (different clusters and disconnection between nodes) (Fig. 4).

### Co-authorship for country

**Fig. 5:**
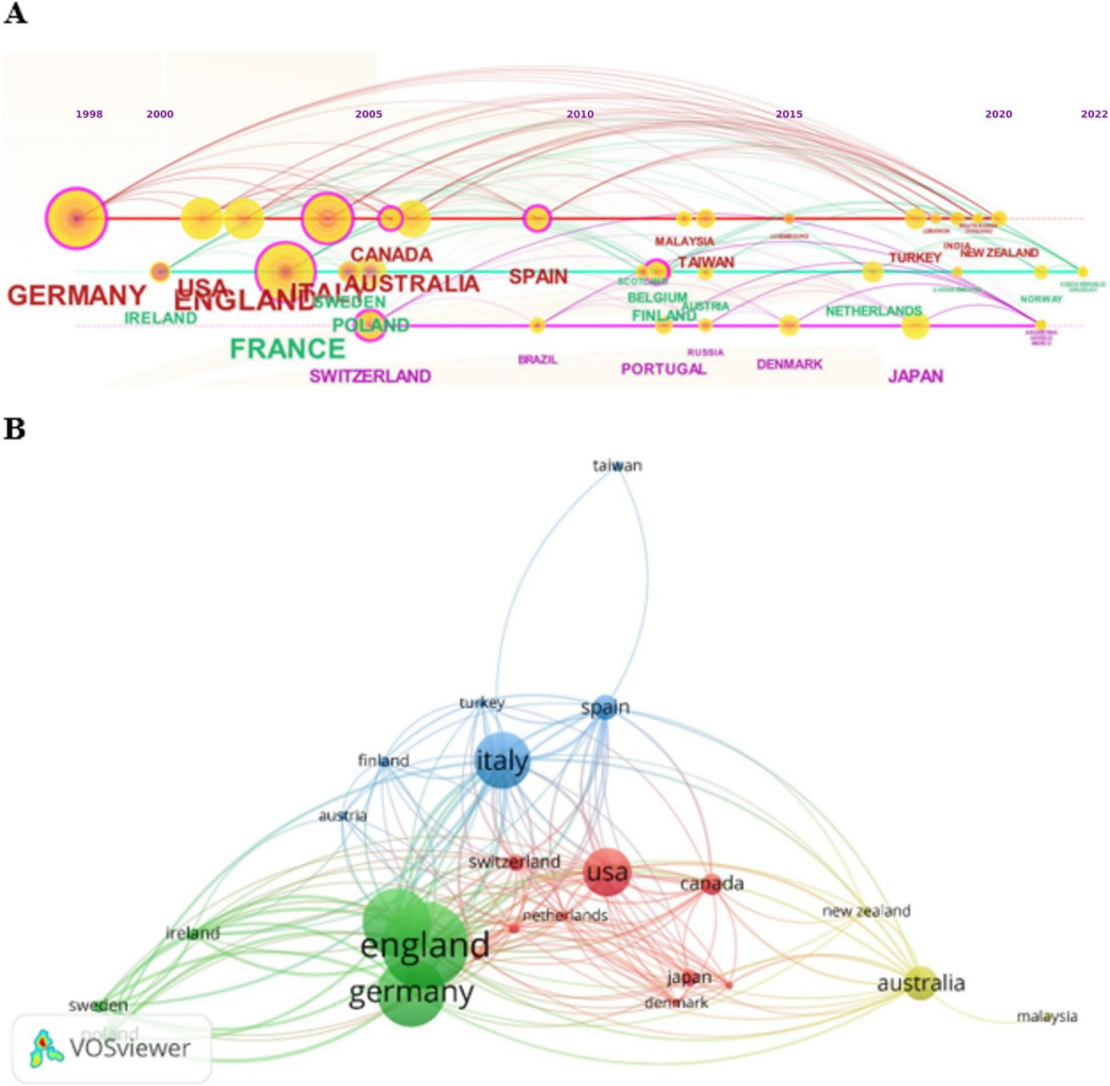
Co-authorship for country. The CiteScope output (**A**) demonstrated annual trends and clusters. Strong connections, especially involving European countries, were more structured in previous years. The VOSviewer analysis (**B**) showed, by considering a minimum of 3 documents for each country, 4 clusters and a total link strength of 369. Nodes in the visualization represent countries, and the size of the node corresponds to the number of papers, with larger nodes indicating a higher paper count. Lines connecting nodes represent citations from different countries, and the thickness of the line reflects the frequency of citations, with thicker lines indicating a higher frequency. Clustering is denoted by color, and nodes of the same color are part of the same cluster

The time-based highlighted that strong connections, particularly among European countries, exhibited a more organized structure in previous years (Fig. 5A). The VOSviewer-based density analysis was performed. Establishing a minimum document threshold of 3 for countries, we calculated the total strength of co-authorship links. Subsequently, countries with the highest link strength were identified. Out of 43 countries, 25 met the specified threshold. Twenty-seventh documents from Italy collected 1619 citations and obtained the highest total link strength (*n* = 80); 41 items from England had 1845 citations and a link strength of 71; 32 from France collected 1408 citations and a strength of 67 (Fig. 5B).

## Discussion

In the context of specialized palliative care, evidence suggests that NIV can be beneficial for patients with advanced respiratory disease [3–7]. Nevertheless, given the complexity of the matter, a thorough investigation is required for various critical aspects of this therapy. We present a knowledge mapping of the scientific output on NIV in palliative care, highlighting key research trends and advancements in the field. This approach can serve as a valuable resource for clinicians and researchers, aiding in the identification of pivotal research and fostering collaboration among experts from various disciplines.

The bibliometric data we gathered demonstrated a consistently positive upward trend over time for both the number of publications and citations. This pattern underscores the increasing focus of the scientific community on addressing a significant healthcare issue. The most cited articles serve as crucial guidance for ALS management. Remarkably, NIV has emerged as a pivotal component in the symptomatic treatment of this clinical condition, enhancing both survival and quality of life. However, managing respiratory insufficiency in these patients is highly complex and demands multidisciplinary expertise. As Dorst et al. [34] suggested, research efforts should be directed toward operational strategies and prognostic factors.

Concerning the quality of the journal, the title that published the highest number of articles on the topic (*Palliative Medicine*, *n* = 12, 6%) is located in the first Q. Most documents were published by journals in the best Qs (Q1 + Q2, 68%). This data represents a strength of the scientific output, although it should be analyzed based on the type of article. Most of the scientific output pertains to qualitative studies. Nevertheless, this finding should not be surprising, given the complexity of the care domain. Obtaining data from healthcare providers and caregivers regarding the quality of care delivered in the field of palliative care is crucial for establishing care programmatic guidelines [35, 36]. Less than one-third of the documents pertain to clinical studies. Among these, the majority consists of retrospective analyses. This finding is not surprising, considering the challenging nature of conducting prospective studies in this clinical domain. However, it allows for the collection of significant datasets to be implemented for retrospective analyses.

Conducting predictive studies using artificial intelligence (AI) and machine learning (ML) would be an intriguing prospect [37]. In this regard, the use of digital technology is recommended by the EAPAC [2]. Among the clinical studies, we highlight the protocol for a multicenter prospective observational cohort study that compares three different oxygenation strategies (high flow oxygen therapy-HFOT alone, NIV alternating with HFOT, and NIV alternating with standard oxygen) in patients admitted to the ICU for acute respiratory failure with a do-not-intubate order [38]. According to the authors, it is important to consider that survival alone should not be the sole objective. Equally significant is the aim to prevent discomfort and restore the patient's quality of life. In a randomized clinical trial involving cancer patients, Nava et al. [27] demonstrated that NIV was more effective than oxygen in alleviating dyspnea and reducing the required doses of morphine in individuals with end-stage disease. It would be advisable to pursue additional high-quality studies on this line of research, given its substantial clinical implications.

Keywords in academic articles are typically chosen to reflect and highlight the main topics, themes, or subjects covered in the content. Our bibliometric study showed that key aspects of palliative approaches, such as communication, withdrawal from therapy, and the quality of care delivered, appear to have received little attention from the authors of the publications. It can reflect a lack of understanding or acknowledgment of the significance of these key aspects in palliative settings. Probably, some dimensions of palliative care, such as communication and the quality of care, may pose methodological challenges in terms of measurement and assessment. Researchers might be deterred by the complexity of studying these areas or may face difficulties in developing standardized tools for evaluation. Furthermore, broader aspects of palliative care, like communication strategies and the psychosocial dimension, might be perceived as less immediate or urgent [39]. Another concern is the lack of papers addressing the inclusion of patient and caregiver experiences in research studies. The analysis of keywords also suggests a significant gap in the methodological aspects of therapy. An important challenge in this field is the definition of therapy protocols that can be tailored to the patient's needs, considering aspects related to the disease, care setting, and resource availability. Finally, we noticed a scarce number of papers discussing palliative care with NIV in COPD patients. This could be explained by the fact that, historically, palliative care focused on the care of dying cancer patients, and in more recent years expanded to include other illnesses, such as ALS. Nevertheless, patients with advanced COPD suffer from breathlessness, fatigue, anxiety, and depression. Consequently, palliative care could be provided according to patients’ needs [40]. We could hypothesize that the scarcity of papers depends on the difficulties in describing the goals of palliative NIV treatment along with the unpredictable illness trajectory of COPD.

The keywords analysis highlights a serious gap concerning the application of advanced research methodologies, including predictive studies using AI and ML. Significantly, another challenge regards the use of telemedicine, remote monitoring, and digital health solutions to enhance the delivery and assessment of NIV in palliative settings. We expect that this gap will soon be filled because the scientific community is inclined towards the integration of these technologies in all medical fields, including palliative care [41–45]. Certain aspects of research will necessarily involve their use in the field of NIV for palliative care (e.g., outcome prediction, therapeutic refinement).

Within the keywords investigation, the correlation between topics is an output of paramount importance [18]. Connections, represented by the distance between keywords, highlight significant gaps in critical aspects, particularly in the relationships between outcome assessment and ventilation strategies [7, 46, 47]. This is evident through the existence of distinct clusters and the disconnection observed between nodes, underscoring the substantial disparities in how these key elements are interrelated or represented within the data. These topics must necessarily be the subject of high-quality studies.

The co-authorship analysis for a country can provide insights into the strength of research collaboration, and the extent of international partnerships (Fig. 5). This strategy is useful for investigating the collaborative networks within the scientific community of a specific country and can contribute to assessments of research productivity and impact on a global scale. In our analysis, we found robust links, particularly among different European countries such as England, Italy, Germany, France, and Spain. Nevertheless, there has been a gap in international collaborations in recent years. The same phenomenon has been highlighted by the analysis of keywords, with a downward trend in co-occurrence (Fig. 4A). Probably, the stronger connections and co-occurrence are due to multicenter studies and guidelines on specific clinical issues such as ALS [24–26]. It is imperative to address and fill this gap focusing the research on different palliative care settings.

## Conclusions

The bibliometric analysis has shed valuable light on the landscape of NIV in palliative care. The discerned patterns and trends not only underscore the growing attention of the scientific community but also highlight the need for continued exploration in this paramount field. There is a call for additional high-quality studies. The multifaceted nature of palliative care, coupled with the intricacies of implementing NIV, prompts the necessity for nuanced investigations into patient acceptance, healthcare provider perspectives, and the integration of NIV with various aspects of palliative care modalities. It becomes evident that the emphasis should extend beyond mere survival metrics. Quality of life, patient comfort, and the holistic restoration of well-being are integral facets that demand further exploration and consideration. Encouraging international collaborations can foster a diverse range of insights, advancing knowledge in the field and stimulating interest and application of this specialized palliative care approach.

## Data Availability

The datasets generated during the current narrative review are available from the corresponding author upon reasonable request.

## Abbreviations

EAPC: European association for palliative care
COPD: Chronic obstructive pulmonary disease
ALS: Amyotrophic lateral sclerosis
NIV: Non-invasive ventilation
ICU: Intensive care unit
WoSCC: Web of science core collection
Q: Quartile
IF: Impact factor
HFOT: High flow oxygen therapy
AI: Artificial intelligence
ML: Machine learning
